# Supplementary material for: Stacking Model‐Based Classifiers for Dealing With Multiple Sets of Noisy Labels
Source: Biom J. 2025 Mar 12;67(2):e70042. doi: 10.1002/bimj.70042 (PMC11898607; doi:10.1002/bimj.70042)
Supplement: Supplementary file 1 — Supporting Information [file BIMJ-67-e70042-s001.zip › density-based-ensemble-model-main/competitors_real_data/netlab-master/netlab-master/help/gpcovar.htm]

Netlab Reference Manual gpcovar


# gpcovar

## Purpose

Calculate the covariance for a Gaussian Process.

## Synopsis

```
cov = gpcovar(net, x)
[cov, covf] = gpcovar(net, x)
```

## Description

`cov = gpcovar(net, x)` takes
a Gaussian Process data structure `net` together with
a matrix `x` of input vectors, and computes the covariance
matrix `cov`. The inverse of this matrix is used when calculating
the mean and variance of the predictions made by `net`.

`[cov, covf] = gpcovar(net, x)` also generates the covariance
matrix due to the covariance function specified by `net.covarfn`
as calculated by `gpcovarf`.

## Example

In the following example, the inverse covariance matrix is calculated
for a set of training inputs `x` and is then
passed to `gpfwd` so that predictions (with mean `ytest` and
variance `sigsq`) can be made for the test inputs
`xtest`.

```
cninv = inv(gpcovar(net, x)); 
[ytest, sigsq] = gpfwd(net, xtest, cninv);
```

## See Also

`gp`, `gppak`, `gpunpak`, `gpcovarp`, `gpcovarf`, `gpfwd`, `gperr`, `gpgrad`

---

**Pages:**
Index


---

Copyright (c) Ian T Nabney (1996-9)
